# Serosurvey and Molecular Detection of *Toxoplasma gondii* in Dogs in Rural Areas of Kazeroun District, Fars Province, Southern Iran

**DOI:** 10.1155/2021/4499086

**Published:** 2021-12-15

**Authors:** Zahra Rezaei, Ali Zeighami, Reza Shahriarirad, Amirhossein Erfani, Mohammad Rastegarian, Nasir Arefkhah, Bahman Pourabbas, Fariba Ghorbani, Bahador Sarkari

**Affiliations:** ^1^Professor Alborzi Clinical Microbiology Research Center, Shiraz University of Medical Sciences, Shiraz, Iran; ^2^Student Research Committee, School of Medicine, Shiraz University of Medical Sciences, Shiraz, Iran; ^3^Cellular and Molecular Research Center, Yasuj University of Medical Sciences, Yasuj, Iran; ^4^Department of Parasitology and Mycology, School of Medicine, Shiraz University of Medical Sciences, Shiraz, Iran; ^5^Basic Sciences in Infectious Diseases Research Center, Shiraz University of Medical Sciences, Shiraz, Iran

## Abstract

*Background. Toxoplasma gondii* is an intracellular protozoan parasite responsible for systemic disease in a wide range of warm-blooded animals. The current study is aimed at evaluating the prevalence of *Toxoplasma* infection in dogs, using serological and molecular methods in rural areas in Kazeroun Township, Fars province, southern Iran. *Methods.* Blood samples were obtained from 60 clinically healthy dogs with an age range of 1 to 7 years in three rural areas of Fars province, southern Iran. Sera and buffy coats were used to assess the *T. gondii* infection using both modified agglutination test (MAT) and real-time PCR. *Results*. Antibodies against *T. gondii* were detected in 5 out of 60 (8.3%) dogs by the MAT method, and *T. gondii* DNA was detected in 17 out of 60 (28.3%) studied animals. There was no significant association between sex and seropositivity to *Toxoplasma* (*p* > 0.05). Fair agreement (kappa = 0.27) was seen between molecular and serological findings where three dogs with positive serological results had a positive molecular test. *Conclusion.* Findings of the present study show a relatively high prevalence of *T. gondii* infection in dogs in rural areas in Fars province, southern Iran. Finding the parasite genotype in dogs deserves further study.

## 1. Introduction

Toxoplasmosis is one of the most common zoonotic parasitic infections which has a high prevalence in human communities [1, 2]. Infection mainly occurs through the consumption of water, food, or soil contaminated with oocytes excreted in cat feces. In addition, the infection can occur by using raw or undercooked meat, containing tissue cysts [2]. It is estimated that 30-50% of the world's population is infected with *T. gondii*, and more than one-third of infected individuals remained asymptomatic [3, 4].

The greatest concern of *T. gondii* infection is in pregnant women who get the infection for the first time since it can be transmitted to the fetus and causes abortion or fetal abnormalities such as ocular or neurological disorders [4]. Toxoplasmosis may also be fatal in immunocompromised individuals because of cerebral toxoplasmosis [5].

Although dogs do not excrete *T. gondii* oocysts in their feces and are not also consumed for foods in most countries, they can serve as a mechanical transmitter [2, 6]. Since dogs are accustomed to rolling in cat feces, they can transmit the infection to humans via their fur [6, 7].

Previous studies have shown that the prevalence of *Toxoplasma* infection among dogs varies in different regions of the world and even within the same country. It has been reported that the prevalence of *Toxoplasma* infection among dogs is 16 to 58% in America [8, 9], 21 to 68% in Asia [10, 11], 23 to 37% in Europe [12, 13], 19 to 25% in Africa [14, 15], and 10 to 31% in Oceania [16, 17]. Nothing is known about the prevalence of toxoplasmosis in dogs from the rural area of Fars province. Hence, the current study is aimed at evaluating the prevalence of *Toxoplasma* infection in dogs using serological and molecular methods in this region.

## 2. Materials and Methods

### 2.1. Study Area and Sample Collection

Blood samples were obtained from 60 clinically healthy dogs with an age range of 1 to 7 years in three villages: Sarmashhad, Tole-e Saman, and Hosseinabad in Kazeroun County, Fars province, southern Iran. Ten mL of blood samples were collected by venipuncture of the cephalic vein from restrained dogs in tubes containing K3-EDTA. Plasma and buffy coats were separated and frozen at -20°C for further serological and molecular tests. The study was approved by the Ethical Committee of Shiraz University of Medical Sciences (Ref. No. IR.SUMS.MED.REC.1398.174).

### 2.2. Modified Agglutination Test (MAT)

Sera were tested for the presence of IgG against *T. gondii,* using the inhouse modified agglutination test (MAT) according to the method described by Desmonts and Remington [18]. Formalin-fixed tachyzoites of *Toxoplasma* were used as antigens. Serum samples were pretreated with 2ME for IgM elimination and diluted two-fold (1 : 20 to 1 : 160) in PBS buffer. Antibody titers of ≥1 : 20 were considered positive.

### 2.3. Detection of *T. gondii* by the Molecular Method

Buffy coats were digested in lysis buffer, containing 0.5% Tween 20, 0.5% Nonidet P-40, 10 mM NaOH, 10 mM Tris (pH 7.2), and 320 mg proteinase K and placed at 56°C for 24 hours and then boiled for 10 minutes. Then, DNA was extracted using the simplified phenol-chloroform extraction method using lysate followed by ethanol precipitation and resuspension in 50 *μ*l of TE (Tris-EDTA) buffer. The real-time PCR was performed using forward and reverse primers targeting a 35-copy number of B1 gene which amplifies a 130 bp region of *T. gondii* [19]. Real-time PCR was performed, using a mixture containing 50 ng of template DNA, 2x SYBR Green PCR Master Mix (Applied Biosystems, US), and 0.5 *μ*M of each primer in a final volume of 20 *μ*l. The reaction was carried out using the StepOnePlus real-time PCR instrument (Applied Biosystems, US) as follows: 10 min hot start at 95°C, 35 cycles at 95°C for 10 s, 68°C for 30 s, and 72°C for 30 s. A melting curve analysis was carried out after the completion of the amplification. Negative control (no template) and positive control (DNA isolated from the *Toxoplasma* tachyzoites) were included in the assay.

### 2.4. Statistical Analysis

Results of the molecular and serological tests, as well as the data related to age, sex, and sampling region, were statistically analyzed, using SPSS software (version 20, IBM Inc., USA) to determine any association between the variables.

## 3. Results

Samples were collected from 60 dogs, of which 23 (38.3%) were from SarMashhad, 13 (21.7%) were from Hosseinabad, and 24 (40%) from Tole-e Saman village. Of the 60 studied dogs, 35 (58.3%) were male and 25 (41.7%) were female. The age range of the dogs was 1 to 7 years with the highest frequency of 2 years old with 13 cases (38.2%), followed by 3 years with 9 (26.5%) cases. Overall, the prevalence of *Toxoplasma* infection, using both serological and molecular methods, in the studied dogs was 31.67%.

Antibodies to *T. gondii* were detected in 5 (8.3%) out of 60 dogs by MAT. *T. gondii* DNA was detected in 17 (28.3%) out of 60 dogs, of which 6 were from SarMashhad, 4 from Hosseinabad, and 7 from Tole-e Saman. Of the 17 dogs whose molecular test was positive, 9 were female, and 8 were male. Fair agreement (kappa = 0.27) was seen between molecular and serological findings where three dogs with positive serological results had a positive molecular test too.  Table 1 shows the characteristics of evaluated dogs and relative prevalence of *Toxoplasma* infection.

## 4. Discussion

The overall frequency of *Toxoplasma* infection among stray dogs in rural areas of Kazeroun district in Fars province in southern Iran was found to be 31.7%. Previous studies have shown different seroprevalences of *Toxoplasma* infection among dogs in a different region of Iran: 46.67% in Ahwaz [20], 10.7% and 35% in Hamedan [21, 22], and 22.47% in Tehran [23]. This difference in seroprevalence is due to differences in the population of dogs studied and kind of the serological tests used for the study, cutoff points, and various environmental and geographical conditions of the study area. Prevalence has been higher in studies that have used the ELISA system [20–23]. It has been documented that the ELISA is more sensitive than MAT for the detection of anti-*Toxoplasma* antibodies [24, 25]. The seroprevalence obtained in the current study (8.3%) was closed to the study performed by Gharekhani et al., with the same serological method and the population of the studied dogs [22]. However, the study area of Gharekhani et al. was different from the one in the current study. There is no information about the food consumed by dogs and also the prevalence of the infection in cats in this region. Also, there is no serosurvey of *Toxoplasma* infection in pregnant women in this region.

In a study by Arefkhah et al., the prevalence of *Toxoplasma* infection in children in the Sarmashad region, where the recent study has been conducted, was investigated. The findings of the study showed a 3.8% prevalence of *Toxoplasma* in this particular group of people in the community [26].

In the current study, the prevalence of the *Toxoplasma* infection was higher using the PCR method. There were twelve dogs with a positive result of PCR but negative MAT. The difference between the PCR and serological results in the current study might be attributed to the cut off point titer in MAT, removal of IgM in MAT procedures, immune status and age of the dogs, or the *T. gondii* strain [27]. As shown in previous studies, positive PCR results are mostly associated with acute infection, when the parasite is in the blood, while stimulation of the immune system and antibody production occurs after encystations of the parasite in the tissues [28].

In previous studies conducted by the researchers of the present study, same samples taken from the dogs examined in this study have been evaluated for *Neospora* and *Leishmania* infections [29, 30]. Comparison of the findings of the present study with previous studies shows that only one case of dogs infected with *Toxoplasma* was also positive for *Neospora* infection. The results were similar for *Leishmania*, and only a small percentage (3.57%) of dogs infected with *Leishmania* were also infected with the *Toxoplasma*. The main reason for this difference in the prevalence of infection with these parasites in the studied dogs is related to the transmission route of the parasite, which is entirely different for *Leishmania* and *Toxoplasma* and somewhat different for *Neospora*. Thus, the frequency of coinfection with these parasites in the studied dogs was low.

There was no significant association between the sex of dogs and prevalence of the *Toxoplasma* infection in the current study [31, 32]. This finding is consistent with the findings of previous studies. According to Dubey and Beattie, *T. gondii* infection in free-ranging carnivores could be an indirect predictor of parasite infection across the regions [1]. Thus, the relatively high prevalence of *Toxoplasma* infection in dogs in the current study indicates a high prevalence of infection in other animals as well as the human population in the studied area.

## 5. Conclusion

The results of the present survey show a relatively high prevalence of *T. gondii* infection among dogs and suggest a need for a larger study on dogs and also a survey of the infection in cats and humans to identify the importance of the dogs in the epidemiology of *Toxoplasma* infection.

## Figures and Tables

**Table 1:** Characteristics of evaluated dogs and relative prevalence of *Toxoplasma* infection, using serological and molecular methods, in rural areas in Fars province, southern Iran.

	Frequency (no.)	Percentage (%)	Positive for anti-Toxoplasma antibodies		Positive for *Toxoplasma* DNA		*p* value
			Frequency (no.)	Percent (%)	Frequency (no.)	Percent (%)	
Sex							0.34
Male	35	58.3	2	5.7	10	27.1	
Female	25	41.7	3	12	7	28	
Age (year)							0.06
0-2	16	26.6	2	12.5	8	50	
3-4	26	43.4	2	5.6	6	23.1	
5-6	13	21.7	1	7.6	2	15.4	
>6	5	8.3	0	0	1	20	
Place							0.09
SarMashhad	23	38.3	1	4.3	8	34.8	
Hosseinabad	13	21.7	3	23.1	4	30.8	
Tolesaman	24	40.0	1	4.2	5	28.3	

## Data Availability

The datasets generated and analyzed during the current study are available in the table. The quantitative data used to support the findings of this study are available from the corresponding author upon request.
